# Protocol for a randomized controlled trial to reduce pediatric anesthesia emergence delirium by titration of sevoflurane anesthesia using brain function monitoring

**DOI:** 10.1186/s13063-023-07785-0

**Published:** 2023-11-16

**Authors:** Yasuyuki Suzuki, Kiyoyuki W. Miyasaka, Kuniyoshi Hayashi, Osamu Takahashi, Yasuko Nagasaka

**Affiliations:** 1 National Center for Child Health and Development, Tokyo, Japan; 2 Tokyo Women’s Medical University, Tokyo, Japan; 3 St. Luke’s International University, Tokyo, Japan; 4 Kyoto Women’s University, Kyoto, Japan

**Keywords:** Pediatric anesthesia, Emergence agitation, Emergence delirium, EEG, Sevoflurane, Randomized controlled trial

## Abstract

**Background:**

Emergence agitation or emergence delirium is a common complication of unknown etiology in pediatric anesthesia. Pediatric anesthesia emergence delirium (PAED) has been reported most commonly in younger children and may occur in about 30% of children up to 5–6 years old. Exposure to anesthetic agents may contribute to PAED, and we hypothesized that a management strategy to minimize exposure to volatile anesthetics may reduce PAED. Electroencephalography (EEG) signatures captured and displayed by brain function monitors during anesthesia change with concentration of sevoflurane and level of unconsciousness, and these EEG signatures may be used to inform titration of anesthetics.

**Methods:**

A single-center, parallel-group, two-arm, superiority trial with a 1:1 allocation ratio will be performed to compare the incidence of PAED following standard sevoflurane anesthesia (maintained at 1.0MAC) and EEG-guided anesthesia (minimum concentration to sustain surgical anesthesia as determined by monitoring of EEG signatures). Participants between 1 and 6 years of age undergoing surgical procedures involving minimal postoperative pain will be randomly assigned to receive standard (*n* = 90) or EEG-guided (*n* = 90) anesthesia. PAED score will be assessed by a blinded observer in the PACU on arrival and after 5, 10, 15, and 30 min.

**Discussion:**

Anesthesia management with proactive use of brain function monitoring is expected to reduce exposure to sevoflurane without compromising surgical anesthesia. We expect this reduced exposure should help prevent PAED. Routinely administering what may be considered standard levels of anesthetic such as 1.0 MAC sevoflurane may be excessive and potentially associated with unfavorable sequelae such as PAED.

**Trial registration:**

Japan Registry of Clinical Trials (jRCT) jRCTs032210248. Prospectively registered on 17 August 2021.

## Administrative information

Note: the numbers in curly brackets in this protocol refer to SPIRIT checklist item numbers. The order of the items has been modified to group similar items (see http://www.equator-network.org/reporting-guidelines/spirit-2013-statement-defining-standard-protocol-items-for-clinical-trials/).

**Table:** 

Title {1}	Randomized controlled trial to reduce pediatric anesthesia emergence delirium by titration of sevoflurane anesthesia using brain function monitoring.
Trial registration {2a and 2b}.	Japan Registry of Clinical Trials, jRCTs032210248. Registered August 17, 2021. https://jrct.niph.go.jp/en-latest-detail/jRCTs032210248
Protocol version {3}	Protocol version 1.6, January 31, 2022.
Funding {4}	Unfunded, with material support (provision of study equipment) from Masimo Corporation.
Author details {5a}	1. National Center for Child Health and Development, Japan 2. St. Luke’s International University, Japan 3. Kyoto Women's University, Japan 4. Tokyo Women’s Medical University, Japan
Name and contact information for the trial sponsor {5b}	This is an investigator-initiated clinical trial conducted by independent investigators. Kiyoyuki W Miyasaka (kmiyasaka@me.com) is the lead investigator.
Role of sponsor {5c}	The design of the study, collection, analysis, and interpretation of data, as well as writing of manuscripts, will be conducted independently by the authors. As a condition of the agreement to provide study equipment, Masimo Corporation will be allowed to review and comment on any manuscripts or abstracts that result from this study prior to submission for publication. However, the agreement explicitly states that the authors are under no obligation to implement any of Masimo’s suggested revisions.

## Introduction

### Background and rationale {6a}

Emergence agitation or emergence delirium is a common complication in pediatric anesthesia. Pediatric anesthesia emergence delirium (PAED) has been reported most commonly in younger children and may occur in about 30% of children up to 5–6 years old [1]. PAED is characterized by behaviors such as restlessness, inconsolability, and non-purposeful movements [2]. The child may cry and thrash about with unusual intensity, risking injury to self as well as accidental removal of catheters and drains. PAED does not last long, resolving in an average of 14±11 min (range 3–45 min), but is clinically significant as it has also been reported to require pharmacological intervention in 52% of cases [3].

The PAED score was developed and validated to quantitatively evaluate this phenomenon [2] and is widely used in the literature [4]. A PAED score of 10 or greater indicates the presence of emergence delirium. Some behaviors associated with PAED may also characterize patients in pain, so it is important to ensure adequate analgesia when evaluating emergence delirium.

The exact etiology of PAED remains unknown. However, it occurs following exposure to anesthetic agents, which modulate and disrupt normal neural activity in the brain. Electroencephalography (EEG) signatures are known to vary with exposure to anesthesia, and brain function monitors that display EEG and EEG-derived indices may be used to guide anesthesia management [5]. Intraoperative burst suppression, which can occur during deep states of anesthesia, is associated with postoperative delirium in adults [6, 7]. In elderly patients, titration of anesthetic agents using EEG has been reported to reduce postoperative delirium [8].

In the pediatric population, a prior study showed intervention with brain function monitoring can significantly reduce sevoflurane exposure (from 2.1 to 1.7%) within a range of nominally normal EEG index values (BIS 60–40), but with an inconclusive effect on preventing PAED [9]. In this trial, we will interpret the EEG signal directly in an attempt to further reduce and minimize sevoflurane exposure and determine its effect on PAED.

### Objectives {7}

We hypothesized that minimizing exposure to sevoflurane through proactive use of brain function monitoring may decrease the incidence of PAED. The difference between groups in the proportion of patients with a maximum PAED score ≥10 in the PACU was specified as the primary outcome.

Differences between groups in maximum PAED score (when treated as a continuous variable), exposure to anesthetic drugs, EEG waveform characteristics, and computed EEG indices (such as Patient State Index (PSi) and Suppression Ratio (SR)) were specified as secondary outcomes.

### Trial design {8}

This randomized controlled trial is a single-center, parallel-group, two-arm, superiority trial with a 1:1 allocation ratio.

## Methods: participants, interventions, and outcomes

### Study setting {9}

The study will be conducted at the National Center for Child Health and Development, an academic pediatric medical center located in Tokyo, Japan.

### Eligibility criteria {10}

Patients aged ≥ 1 to < 6 scheduled for elective surgical procedures under general anesthesia will be screened for eligibility. Patients undergoing procedures expected to be at least 30 min long involving minimal postoperative pain will be included. Those with neurological or developmental conditions that may hinder the evaluation of PAED will be excluded, as will procedures or other factors that may interfere with proper sensor placement and brain function monitoring (e.g., head and neck surgery, prone position, skin conditions).

### Who will take informed consent? {26a}

Patients who meet the above criteria will be approached by a member of the research team following their preoperative visit to the anesthesia outpatient clinic, and written consent will be obtained from a parent or legal guardian.

### Additional consent provisions for collection and use of participant data and biological specimens {26b}

No biological specimens will be collected. Any ancillary studies using participant data will need to be approved by the institutional review board.

### Interventions

#### Explanation for the choice of comparators {6b}

For sevoflurane, 5% is the maximum induction dose indicated on the package insert. In the control group, anesthesia will be induced using 5% sevoflurane with 66% nitrous oxide in oxygen. Atropine 0.01mg/kg, rocuronium 1mg/kg, and fentanyl 2μg/kg will be given per institutional routine as soon as intravenous access is established. Conventional wisdom in the anesthesia community suggests that at least 1.0MAC should be maintained to prevent unintentional awareness [10] in the absence of brain function monitoring. After intubation, sevoflurane will be maintained at 1.0MAC (approximately 2.5% for this age group) in an air/oxygen mixture. Intraoperative analgesia will be provided with remifentanil 0.5μg/kg/min in addition to procedure-appropriate nerve blocks and/or caudal epidural and/or local infiltration anesthesia using up to 1ml/kg of 0.2% ropivacaine. Fentanyl 1–2 μg/kg and acetaminophen 15 mg/kg (7.5 mg/kg if <2 years of age) may be given near the end of the procedure for additional postoperative analgesia. The brain function monitor screen will be kept out of view to prevent any influence on anesthesia management in this group.

#### Intervention description {11a}

In the EEG group, sevoflurane will be titrated based on EEG findings to the minimum necessary level to induce and maintain surgical anesthesia. The targeted EEG finding is a sustained alpha and delta-dominant EEG waveform and spectrogram pattern that is characteristic of GABAergic anesthetics such as sevoflurane. At lower concentrations of sevoflurane, the frequency of the alpha band increases and begins to intermittently dissipate into higher-frequency bands, while maintaining the delta band. At this point, sevoflurane may be increased to reestablish the alpha-delta dominant pattern. At higher concentrations of sevoflurane, the frequency of the alpha band decreases and it may begin to merge with the delta band. Burst suppression may also be observed. If so, sevoflurane concentration is likely excessive and may be reduced.

Management other than sevoflurane will be per institutional routine as described for the control group. A single researcher extensively trained in brain function monitoring (KWM) will induce and maintain anesthesia for all subjects. A separate attending anesthesiologist (not part of the research team) will supervise each case with the authority to take over and abort the research protocol if there is any concern for patient safety.

#### Criteria for discontinuing or modifying allocated interventions {11b}

The intervention may be discontinued if there are changes in the scheduled procedure that alter the participant’s eligibility, such as the addition of surgical procedures that may involve significant postoperative pain. The intervention may be modified and/or discontinued at any point if the researcher or attending anesthesiologist decides proceeding per protocol poses a concern for patient safety.

#### Strategies to improve adherence to interventions {11c}

The entire intervention and observations will be conducted directly by members of the research team to ensure adherence to protocol.

#### Relevant concomitant care permitted or prohibited during the trial {11d}

There are no restrictions on concomitant care and interventions during the trial.

#### Provisions for post-trial care {30}

Ancillary and post-trial care, including care for any harm resulting from trial participation, will be provided as part of routine postoperative and post-anesthesia care. No additional compensation is planned, as the trial does not involve risks beyond what may result from routine general anesthesia management.

### Outcomes {12}

Primary outcome: difference between groups in the proportion of patients with a maximum PAED score ≥ 10 in the PACU on the day of surgery.

Secondary outcomes: difference between groups in maximum PAED score in the PACU on the day of surgery, exposure to sevoflurane (maximum concentration, mean concentration, total MAC-hours exposure), EEG indices (mean PSi during surgery, duration of SR>0), and EEG waveform characteristics (frequency power spectrum) on the day of surgery.

### Participant timeline {13}

**Table:** 

	Study period				
	Enrolment	Allocation	Intervention	Observation	Close-out
Setting	Anesthesia outpatient clinic	Surgery date confirmed	Operating room	PACU	Post-operative rounds or discharge to home
Timepoint	−t1	0	t1–t2 (day of surgery)		t3 (24±6 h from t2)
Enrolment:					
Eligibility screen	X				
Informed consent	X				
Allocation		X			
Interventions:					
Control			X		
EEG			X		
Assessments:					
Medical record	X				X
PAED score			X	X	
EEG			X	X	
Adverse events			X	X	X

### Sample size {14}

Based on a previous study [11], we calculated that 87 subjects per group powered the study to detect a clinically significant reduction in the incidence of PAED (conservatively, reduced by half from 38.3 to 19.2%) using the chi-squared test at an alpha of 0.05 and beta of 0.8. The target sample size was set at 90 subjects per group to account for a small number of dropouts.

### Recruitment {15}

The planned study site is one of the largest pediatric centers in Japan, with approximately 4500 surgical cases per year. Accrual of the target sample size is expected to be possible in about one year.

## Assignment of interventions: allocation

### Sequence generation {16a}

Enrolled patients will be randomly assigned to either the “Control” or “EEG” group by an online computerized service that uses permutated block randomization (block size = 4) stratified by year of age to assign an intervention. The service also returns a unique six-character alphanumeric code that will be used as an identifier for patients within the study.

### Concealment mechanism {16b}

The sequence of patient allocation is generated on request by computer and remains concealed until assigned.

### Implementation {16c}

An online computerized service will generate the allocation sequence and assign participants to interventions on request. Upon receipt of an eligible participant’s signed consent form, the lead researcher (KWM) will enroll the participant and request an allocation from the online service.

## Assignment of interventions: blinding

### Who will be blinded {17a}

The trial participant and parents/guardians, as well as the researcher assigned to assess PAED score (YS, co-lead researcher, who has over 40 years of clinical experience in pediatric anesthesia), will not be informed of intervention assignment and will remain blinded until completion of outcome observations. Due to the nature of the intervention, the researcher performing the intervention (KWM, lead researcher, who underwent expert training for brain function monitoring) and other anesthesia providers directly involved in the trial participants’ care cannot be blinded.

### Procedure for unblinding if needed {17b}

Unblinding and disclosure of intervention assignment are permissible after the conclusion of outcome observations for the participant, or at any point if needed to address concerns for patient safety. Unblinding during the trial is not thought to be necessary by design as those performing the intervention cannot be blinded. If unblinding of the outcome observer does occur during the trial, the participant will be excluded from subsequent analyses.

## Data collection and management

### Plans for assessment and collection of outcomes {18a}

The PAED score was developed and validated to quantitatively evaluate this phenomenon [2] and is widely used in the literature [4]. A single researcher blinded to the intervention will perform the score assessment to eliminate interrater variability. Anesthesia and operative events (e.g., time and dose of drugs, start and end of surgery) will be recorded to the nearest minute in the electronic anesthesia record. Time on the brain function monitor will be synchronized to the anesthesia record before each case, and all data will be exported and annotated within the same day to reduce the risk of errors including accidental erasure.

### Plans to promote participant retention and complete follow-up {18b}

All study interventions and follow-up conclude during the participant’s perioperative inpatient stay—within 24 ± 6 h of surgery or upon discharge to home, whichever comes first. There is no expected need to retain patients to complete follow-up. For participants who discontinue or deviate from the protocol, ongoing data collection (such as EEG waveforms) will be continued as feasible, but will not be included in the final analysis.

### Data management {19}

All data entry will be performed by the lead researcher, and electronic files will be stored on a password-protected disk image. Paper data entry forms will be stored in a locked file box placed in a restricted staff-only area of the operating room.

### Confidentiality {27}

Personal information of potential and enrolled study participants will remain within the institution’s electronic medical record system before, during, and after the trial. The only personal information that will reside outside the medical record system is each participant’s consent form, which will include the consenting parent or guardian’s name, the patient’s name, medical record number, and unique trial identifier. Collected electronic data (such as EEG) will be labeled with the trial identifier and will not contain personal information. Signed consent forms will be stored under lock and key by the lead researcher for 5 years following completion of the trial as required by Japanese law (the Clinical Trials Act).

### Plans for collection, laboratory evaluation, and storage of biological specimens for genetic or molecular analysis in this trial/future use {33}

Not applicable; no biological specimens will be collected for this study.

## Statistical methods

### Statistical methods for primary and secondary outcomes {20a}

Statistical difference in the proportion of patients with PAED≥10, the primary outcome, will be analyzed using the chi-squared test. Secondary outcomes will be analyzed using the *t*-test for continuous variables, or the chi-squared test for proportions. Differences in EEG signatures will be analyzed using the Multitaper Frequency-Domain Bootstrap Method described by Kim et al. [12, 13].

### Interim analyses {21b}

An interim analysis of the primary outcome by the lead researcher is planned after the recruitment of 90 subjects (50% of target recruitment). The interim results will be shared with other researchers in the trial and the data monitor, with the possibility of early termination of the study if a large effect (*p*<0.025) is observed for the primary outcome.

### Methods for additional analyses (e.g., subgroup analyses) {20b}

Subgroup analyses may be performed by age or surgical factors (e.g., type, length of surgery) using the same statistical methods as the primary and secondary outcomes analyses.

### Methods in analysis to handle protocol non-adherence and any statistical methods to handle missing data {20c}

Subjects with missing/insufficient data or data associated with non-adherence to protocol will not be included in the analysis.

### Plans to give access to the full protocol, participant-level data, and statistical code {31c}

Study participants may request and be granted access to the full protocol. Participant-level data will be deidentified and made public as an appendix to the final results manuscript, or made available on request to the corresponding author. Statistical code for EEG analysis is based on previously published work [12, 13] that is publicly available.

## Oversight and monitoring

### Composition of the coordinating center and trial steering committee {5d}

This is a small single-center study managed mostly by the lead researcher (KWM). The lead researcher and co-lead researcher (YS) responsible for PAED score observations meet before and after each case. A separate researcher not involved in the administration of the study itself (YN) will provide oversight, meeting with the lead researcher as needed. Trial status will be reported regularly to the IRB as required by Japanese law. There is no separate steering committee.

### Composition of the data monitoring committee, its role and reporting structure {21a}

Data monitoring will be performed by an individual familiar with the study protocol not involved in the administration of the study itself. Monitoring will take place after the interim analysis and at the conclusion of the trial via site visits (or remote video conference) with the lead researcher (KWM) and follows a structured reporting format. Findings from monitoring will be reported to the IRB as required by Japanese law.

### Adverse event reporting and harms {22}

Adverse events and other unintended effects are required to be reported to the hospital management, the IRB, and other parties as specified by Japanese law.

### Frequency and plans for auditing trial conduct {23}

Outside of monitoring, audits of trial conduct are not planned for this study.

### Plans for communicating important protocol amendments to relevant parties (e.g., trial participants, ethical committees) {25}

Any protocol amendments will be approved by the IRB and made public via jRCT in accordance with Japanese law. Informed consent documents will be revised and approved if needed to reflect relevant changes.

### Dissemination plans {31a}

Results will be submitted for publication to peer-reviewed journals and presented at academic meetings for dissemination within the medical community. Study status including publication of results will be publicly registered to jRCT in accordance with Japanese law.

## Discussion

If PAED is significantly reduced by the study intervention, we believe this will support our hypothesis that reducing exposure to anesthesia has a preventive effect on PAED and that EEG-guided anesthesia may be used to achieve this effect. If no significant difference is observed in PAED between groups, this may suggest that the as-yet-unknown mechanism of PAED is not strongly influenced by the amount of intraoperative exposure to sevoflurane. Future efforts to prevent PAED may need to focus on other factors, such as properties of emergence (emergence trajectories) and/or choice of anesthetics. However, even if the study intervention has no preventive effect on PAED, reductions in exposure and use of less sevoflurane may have other merits, such as faster time to emergence, shorter PACU stay, and lower environmental impact from waste anesthetic gases. While we do not expect PAED to increase paradoxically as a result of reduced exposure to sevoflurane, analyses of secondary outcomes such as EEG waveform characteristics leading up to emergence may provide additional insights in this case.

This is a small study performed with minimal personnel and resources. The lead investigator thus has multiple roles, controlling most aspects of the study. However, care was taken in the design to ensure the primary outcome is always evaluated by a separate, blinded observer.

## Trial status

The current protocol version is 1.6 (January 31, 2022). Recruitment began on October 13, 2021, and the study is projected to be completed by March 31, 2023.

## Abbreviations

PAED: Pediatric anesthesia emergence delirium
EEG: Electroencephalography
BIS: Bispectral index
MAC: Minimum alveolar concentration
PACU: Post-anesthesia care unit
IRB: Institutional Review Board
jRCT: Japan Registry of Clinical Trials
